# Expression cloning human and rat renal cortex Na/P_i_ cotransporters: behind the scenes in the Murer laboratory

**DOI:** 10.1007/s00424-018-2198-9

**Published:** 2018-09-03

**Authors:** Simona Magagnin, Andreas Werner

**Affiliations:** 1 Biologa Nutrizionista, Casatenovo, Milan, Italy; 2 Institute for Cell and Molecular Biosciences, Epithelial Research Group, Newcastle University, Newcastle, UK

**Keywords:** Sodium-dependent phosphate transporter, Expression cloning, Kidney, Proximal tubule, Solute transport

## Abstract

In the pre-genomic era, the cloning of a cDNA represented a significant achievement, particularly if the gene of interest encoded a membrane protein. At the time, molecular probes such as partial peptide sequences, suitable nucleic acid sequences, or antibodies were unavailable for most proteins and the “sodium-phosphate transporter” was no exception. In contrast, brush-border membrane vesicles and epithelial cell culture experiments had established a reliable set of functional hallmarks that described Na-dependent phosphate transport activity in some detail. Moreover, aspects of hormonal regulation of phosphate homeostasis could be recapitulated in these model systems. Expression cloning elegantly combined functional protein expression in *Xenopus laevis* oocytes with molecular biology to overcome the lack of molecular probes.

## Introduction

The importance of inorganic phosphate (P_i_) to human health and the hormonal feedback loops to maintain homeostasis were well established when the two authors, Simona Magagnin and Andreas Werner, joined the Murer lab in 1991 and 1987, respectively. Detailed studies using brush-border membrane vesicles (BBMVs) from kidney and intestine had established functional characteristics of the “Na-P_i_ cotransporters” such as apparent affinities for P_i_ and Na^+^ as well as the different pH dependence of intestinal and renal transport activities. Moreover, experiments with opossum kidney (OK) cells elegantly confirmed the regulatory impact of hormonal and metabolic factors on Na-dependent P_i_ transport activity [18]. At that point, the molecular identity of the Na/P_i_ cotransporter became a pressing question and different strategies were initiated to identify the protein. Approaches comparing protein signals on 2D gels with or without stimulation of Na-P_i_ transport activity were pursued, though with limited success: The resulting protein pattern was too complex and not reproducible enough to identify differentially expressed signals that would warrant peptide sequencing [26, 35]. In summer 1987, Heini Murer organized a meeting in the Swiss Alps (Fürigen) on epithelial P_i_ transport that was attended by many of the key figures in the field. On this occasion, Ernest Wright and Michael Coady (UCLA) presented the cDNA sequence and primary structure of the intestinal Na/glucose transporter SGLT1 achieved by expression cloning [11]. Michael Coady paid a brief visit to the laboratory in Zürich afterwards and presented us with his winner’s perspective on how easy and straight forward this cloning strategy was. The reality was to prove considerably more tedious and less straightforward than predicted—for example, it took us an entire year to confirm the stunningly clear, first demonstration of Na-dependent P_i_ cotransport in *Xenopus* oocytes injected with rabbit kidney mRNA. Eventually, protein expression in oocytes proved rather straight forward and then became an essential tool in the Murer lab to clone and characterize P_i_ and other solute transporters.

## Expression cloning

**Fig. 1:**
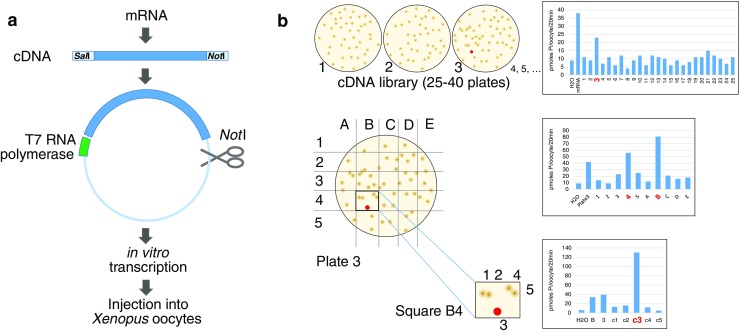
Schematic representation of the expression cloning strategy. **a** Summary of the molecular biology arm of the cloning strategy. Reverse transcription was primed using an oligo-dT-*Not*I adaptor primer. After second strand synthesis, *Sal*I linkers were added, followed by *Not*I restriction digest. The two different “sticky” ends enabled efficient directional cloning—but excluded the functional cloning of cDNAs that contain an internal NotI site (as, for example, human NaPi-IIc). **b** Outline of the screening strategy using functional expression in *Xenopus* oocytes. The left panel explains the steps to successively narrow down the number of clones. Each of the initial plates contained 600–1000 individual bacterial colonies. Sib selection, as exemplified in the second screening step (middle panel), was applied to reduce the number of injections. The right panel shows the uptake results obtained with the RNA synthesized from the related plasmid pools

The expression of proteins in *Xenopus laevis* oocytes was pioneered by John Gurdon (University of Cambridge) who was awarded the 2012 Nobel Prize in Medicine and Physiology for his ground-breaking work [9]. In an application of the original strategy in the mid-1980s, Tasuku Honjo (Kyoto University) and his group injected in vitro-transcribed RNA from a T cell cDNA library and used the oocyte supernatant to stimulate growth and colony formation of B cells. By subdividing the library into smaller and smaller pools, eventually, a single clone was isolated—encoding interleukin 4—that conferred biological activity [24]. This strategy was quickly applied by two groups to the cloning of membrane proteins: Shigetada Nakanishi’s lab (Kyoto University) used electrophysiology to isolate a cDNA that induced membrane depolarization upon administration of substance K in *Xenopus* oocytes [20]. At the same time, Ernest Wright’s group (UCLA) measured the flux of a radioactive glucose analogue to isolate and characterize a clone encoding the Na-glucose cotransporter SGLT1 [11]. These three seminal papers provided the corner stones for the expression cloning strategy using *Xenopus* oocytes that would become the “golden bullet” for the cloning of membrane proteins in the pre-genomic era (Fig. 1) [28].

In essence, the technique involves the injection of a few nanoliters of mRNA into the cytoplasm of oocytes harvested from frogs (*Xenopus laevis*) using a pulled glass capillary. The cells translate the RNA and integrate the resulting protein into the plasma membrane where its function can be analyzed. Initially, poly-A-selected mRNA, often length fractionated using preparative gel electrophoresis, was injected [12, 19]. Interestingly, the mRNAs usually gave robust functional signals despite the Na-phosphate transporter encoding message only representing a minimal fraction of the total mixture. In fact, it is the signal from mRNAs that predicts success or failure of an expression cloning strategy. Once this key experiment proves successful [31], a cDNA library is constructed from the relevant mRNA (Fig. 1a). The mix of clones is then singled out on agar plates, and in a fiddly process using circular nylon membranes, two exact replicas are taken. The bacterial clones from the first replica are then pooled, grown, and the plasmids are extracted. From the plasmid mix, RNA is synthesized in vitro and then tested in *Xenopus* oocytes. The goal is to find one (or more) RNA samples from individual plates (pools of clones) that significantly induce the uptake of substrate (P_i_) over water-injected controls. The second replica filter of the “positive” plate is then cut into about 25 squares containing smaller pools of bacteria and the screening process is repeated. Eventually, single colonies are picked from the square that stimulates uptake and tested for function. With some luck and perseverance, a single clone will eventually be identified that reliably induces the expected function after expression in *Xenopus* oocytes (Fig. 1b).

## “Straight forward and easy”—you wish!

Despite its power, expression cloning remained a daunting task that required cross-discipline methodology and was often done in collaborations with other laboratories. The first attempt in the Murer laboratory to clone the “Na-P_i_ transporter” involved a key collaboration with molecular biologists, Marilyn Moore, Ned Mantei, and Giorgio Semenza at the ETH in Zürich. The cDNA library was prepared in their lab and the resulting in vitro-transcribed RNA was then coded and tested blindly in Heini Murer’s laboratory. Ironically, despite taking all precautions to rule out experimental bias during the screening steps, NaPi-I turned out to be the wrong clone [33]. It was later shown that the alleged P_i_ transporter displayed anion permeability and was related to neuronal vesicular glutamate transporters [1, 29]. NaPi-I did induce phosphate uptake in oocytes, though many of the previously established features of Na-phosphate transport were not convincingly reproduced by NaPi-I [33]. For example, the cloned cDNA was about 0.5 kb shorter than the mRNA fraction that induced highest P_i_ transport in oocytes [31]. Could this be the consequence of poly-A tail shortening or was it a gel artifact? Moreover, minimal amounts of injected NaPi-I RNA saturated Na-dependent P_i_ uptake in oocytes, contradicting observations made with SGLT1 and fractionated mRNA where a linear dose to signal relation was found to 10 ng RNA per oocyte or even higher [31, 33]. Could it be that the oocytes did not tolerate a P_i_ overload and inhibit further expression of the injected RNA? Nobody could tell at that point. To test a putative role of NaPi-I in maintaining P_i_ homeostasis, rabbits were fed high and low P_i_ diets and NaPi-I RNA and protein levels were assessed [6]. To our frustration, no regulatory effect of P_i_ on NaPi-I was detected and it became undeniable that we had put our bets on the wrong clone: In default of a soul, the devil puts up with a fly—or, in our case, a few P_i_ molecules slip through a glutamate transporter if no other substrate is available. Hence, the measured substrate flux was a mere by-product and not the physiologically relevant transport activity of the expressed protein. In retrospect, some of the problems that tricked us into identifying the “wrong” clone have been resolved, others remain enigmatic. The unsolved ones relate to the biology of oocytes, whereas most of the solved problems are of technical nature, and both aspects will be discussed in some detail below.

## The good oocyte, the bad oocyte, and the ugly (and the consequences of mistaking one for the other)

Quality oocytes were key to reliable protein expression and it comes as no surprise that the frogs (South African claw-toed frog, *Xenopus laevis*) and their precious oocytes were at the center of perpetual discussions and concerns in Heini Murer’s lab. Often, the usual “good morning!” was preceded by “how are the oocytes?” and this was a make-or-break my day question. Thousands of injected oocytes and hours of uptake to assess transport were required to narrow down the selection of RNAs from bacterial clones and reliable uptake measurements were essential. “Bad” oocytes had increased Na^+^-independent phosphate permeability and displayed uptake rates that were clearly above controls (water-injected or uninjected oocytes), and indistinguishable from positive signals. Moreover, such batches usually showed large variations between individual cells. As a result, a group of injected “bad” oocytes could contain cells with clearly increased transport activity (real signal or artifact?) as well as oocytes with transport rates close to the negative controls (non-functional RNA or an oocyte receiving little or no RNA during injections?). Overinterpretation of such experiments could easily send the project down the wrong track and it was often safer to hope for better oocytes and repeat the entire experiment than taking a bet. Problems mostly occurred during summer, oocytes had “spots,” were soft to inject, or would not last long enough to show optimal expression. Unfortunately, during all these times, we were unable to establish key determinants that would guarantee a constant supply of “good” oocytes. The influence of a seasonal cycle was discussed, though several observations complicated the picture: The frogs were kept at constant water temperature with a 12-h day/night cycle in a room without windows; moreover, the problems occurred during European summer with frogs imported from South Africa, but also with the ones bred in Germany. The general credo was that happy frogs would give good oocytes and a lot of consideration was given to food, husbandry, and pest control. The oocyte users, Simona Magagnin, Juan Bertran, Daniel Markovich, Manuel Palacin, and Andreas Werner, were also responsible for feeding the frogs and cleaning the tanks. Food pellets were imported from Germany (Horst Kaehler, Hamburg) and the animals were fed twice a week. The water had to be left for chlorine to evaporate for at least a day before filling the aquarium; otherwise, the frogs would develop “red legs”, a skin disease caused by *Aeromonas* bacteria. The frogs’ well-being, their skin, color, and behavior were monitored closely, and often, the nearby veterinary hospital was approached for advice. The frogs were looked after more like pets than lab animals and much of the care was guided by hearsay and exchange with other oocyte labs rather than published guidelines (of which there are many nowadays, for example, https://www.med.hku.hk/images/document/04research/culatr/04_Xenopus_RSPCA%20Guidelines_05.pdf). The less stringent animal protection rules allowed reusing individual frogs as long as we were happy with their oocytes. That implied a surgical procedure to open the skin, fascia, and the underlying muscular layer to harvest the oocytes. Through a small opening in the abdomen, a few ovarian sacs were removed, rinsed in oocyte Ringer’s solution, and further defolliculated with collagenase [31]. Finishing the procedure required a few stiches, and the basic surgical tricks my medical student girlfriend taught me helped with the fiddly task. Despite the pragmatic and often improvised approach we took in isolating the *Xenopus* oocytes, we hardly lost any animals due to operative surgical errors [27]. Interestingly, the frogs never developed wound infections despite minimal effort to keep the surgical tools sterile and the absence of post-operative precautions. Similar observations had prompted Michael Zasloff to investigate the phenomenon that leads to the discovery of a novel family of antimicrobial peptides from frog skin, the so-called magainins [36].

In addition to the often unpredictable nature of frogs and oocytes, the equipment used to inject the cells was—at least initially—primitive and did little to control variability. For the first injections, we used a 50-ml syringe connected to the pulled glass capillary with a narrow silicone tube to which a vacuum (to aspire the RNA) and positive pressure (for injections) was applied. The injected volume was controlled by visually inspecting the meniscus of the RNA solution moving along a stripe of graph paper glued to the back of the capillary. Another matter of concern were impurities in the RNA or sticky material from the injected oocytes that easily blocked the capillary tip. In such a no-win situation, one could either break the tip of the needle with negative consequences to oocyte survival and signal variability or change the capillary and risk losing the precious RNA. With time, the injection equipment significantly improved; nowadays, there are precise, purpose-built oocyte injection systems, and even automated devices are available. Moreover, in the pre-kit era of molecular biology, the RNA preparations often contained traces of salts or organic solvents and the purity of the injected RNA significantly affected oocyte performance. Despite the many technical improvements, the rather varied expression of NaPi-II proteins in individual oocytes persisted and is likely to depend on the particular NaPi-II isoform (species of origin) and how efficiently the particular protein is integrated into the oocyte membrane.

## Molecular biology

At the beginning of the 1990s, molecular biology was still perceived as a scientific discipline in its own right; the development of specialized kits for complex procedures had only just begun. One of the early kits, the unidirectional cDNA library construction system, SuperScript from Gibco-BRL, proved a real game changer: the protocol yielded high-quality cDNA libraries with 10^5^ clones or more (far exceeding the number of clones that could realistically be screened) without the requirement of advanced molecular biology skills (Fig. 1b). Another substantial help were kits to synthesize RNA using T7 and SP6 RNA polymerases including a Cap analogue, the mMessage mMachine from Ambion becoming the favored option; however, the latter kits were not available for the original cloning of rat and human NaPi-II (NaPi-2 and NaPi-3).^1^ Though, the challenges did not end with the successful isolation of a single, functional cDNA. Sequencing of kilobases of DNA posed a significant challenge, even with the reliable Sanger method. To illustrate the scale of the task, Heini Murer spent a sabbatical semester 1989 in Joseph Handler’s lab at the NIH (National Institute of Health, Bethesda) sequencing the renal aldose reductase cDNA of approximately 1.3 kilobases (kb) [7]. With polyacrylamide-urea sequencing gels resolving at best 400 bases and primers costing about 25 times of today’s price, sequencing was time consuming and expensive. The method of choice at the time was to chop the insert into small fragments using two or three frequently cutting restriction enzymes. The resulting pieces were cloned and Sanger sequenced using vector-encoded primer binding sites. The resulting patchwork of sequences was complemented with few sequencing reactions using specific primers. Assembling the snippets and establishing a consensus sequence with an open reading frame was the next task, achieved by pattern recognition. For lack of computer-driven algorithms, consensus sequences were combined by eye and intuition, sliding lines of A, T, C, and G along each other, and searching for noticeable sequence motifs on different fragments to establish an overlap. A compression on a sequencing gel, caused by CG-rich regions or repeats, could easily shift the reading frame and it only took a compensatory misinterpretation downstream of the original error to get back in frame. Such a mistake resulted in a stretch of erroneous amino acids that would be easily spotted if homologous sequences had been available. Though, without reference, the possibility of major sequencing errors was real: Whereas the originally published sequences from rat (NaPi-2/Slc34a1) and human (NaPi-3/SLC34A1) were correct, the flounder transporter (NaPi-5/Slc34a2) cloned in collaboration with the Kinne laboratory (Dortmund) was published with a stretch of 98 amino acids in the wrong reading frame. The mistake was only recognized and corrected 2 years later [15, 34].

## NaPi-IIa, Slc34A1

The cloning of NaPi-I, as disappointing the outcome may have been, meant that the crucial procedures in expression cloning such as cDNA library construction, in vitro transcription, and the functional assay in *Xenopus* oocytes had been extensively tried and tested. For the novel expression cloning approach, the entire experimental processes including the molecular biology techniques were performed in the Murer lab. This endeavor was helped by novel kits which facilitated the molecular biology arm of expression cloning significantly. In addition, it was a fortunate coincidence that a second transporter-oriented laboratory in Zurich with Bruno Hagenbuch, Bruno Stieger, and Peter Meier embarked on the expression cloning of bile acid transporters at the same time we started hunting for NaPi-II [10]. One positive input was certainly the stimulating exchange about oocytes and technical pitfalls, more practically, Peter Meier’s group had an electroporator that gave fabulous transfection rates and significantly improved the coverage of our cDNA libraries. Another fortunate coincidence provided us with an excellent internal control to monitor every aspect of the screening process: Juan Bertran and Manuel Palacin, at that time guests in the Murer laboratory, had just characterized an amino acid transport activity with renal mRNA. The elicited flux of ^3^H arginine was consistently high and virtually without variability between individual oocytes [5]. Hence, we screened our human kidney cDNA library for both phosphate and arginine transport and the latter proved an excellent indicator for the quality of the oocytes and confirmed the biological activity of the injected material. Thanks to the collaborative atmosphere in the Murer lab and the fact that other cloning projects ran in parallel, the screening of both rat and human kidney cDNA libraries progressed without major events. At the end of the process, we not only isolated clones for rat and human NaPi-IIa (then NaPi-2 and NaPi-3) [16] but also, quasi as a bonus, the human amino acid transporter rBAT [4]. Moreover, the obvious sequence similarities between the rat and the human clone helped the assembly of the final sequences and suggested a distinct structure-function relationship.

## Got a clone, now what?

**Fig. 2:**
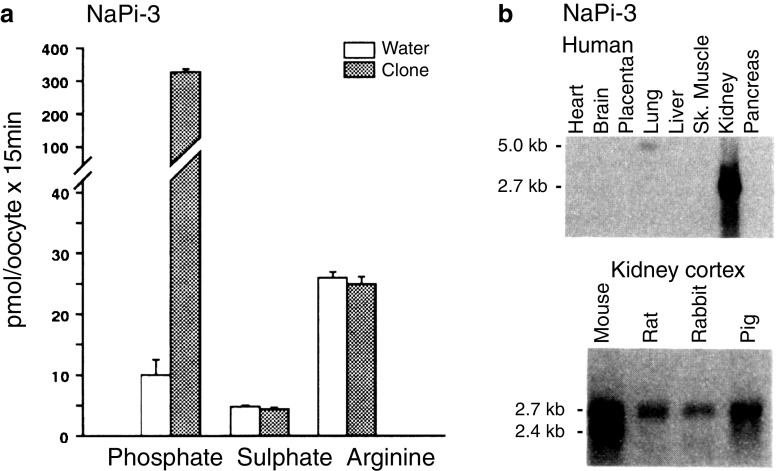
Characterization of the human cDNA. **a** Functional test of in vitro-transcribed RNA expressed in oocytes. The expressed transporter is specific for P_i_ and does not accept neither sulfate nor arginine. **b** Northern blot including RNA from different human tissues (top) or RNA isolated from renal cortex of different species (bottom). The faint band of approximately 5 kb in human lung is likely to reflect the highly expressed SLC34A2 (intestinal) isoform. Mouse appears to express two different renal isoforms derived from alternative polyadenylation. The figure is composed of original material published by Magagnin et al. [16]

**Fig. 3:**
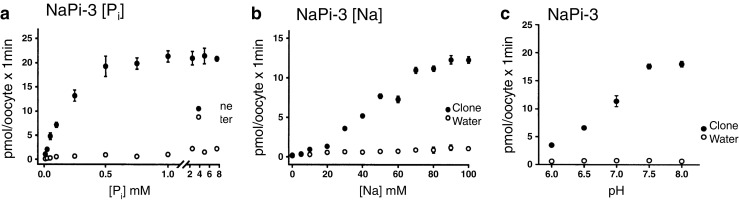
Functional characterization of NaPi-3, the human Na-Pi cotransporter. In vitro*-*transcribed RNA was expressed in Xenopus oocytes and transport activity measured by radiotracer flux under different experimental conditions. **a** Varying concentrations of P_i_ were included and transport showed hyperbolic saturation kinetics indicating that one Pi molecule was transported per cycle (apparent K_m_ 0.170 mM). **b** Na kinetics showed a sigmoidal shape pointing to cooperative binding of at least 2 Na^+^ ions per transport cycle. **c** Transport showed pronounced pH dependence with maximal transport rates at neutral pH and inhibition in acidic conditions. Figure from original material published by Magagnin et al. [16]

After barking up the wrong tree with NaPi-1, we were cautious with the introduction of the “real” NaPi transporters and performed a series of experiments to establish the physiological relevance of the isolated genes. From the start, the P_i_ flux induced by the cloned cDNA/RNA was impressive; no sign of “P_i_ overload” was observed (Fig. 2a). Moreover, transport activity was highest at neutral pH and decreased when the uptake solution was acidified, much like the P_i_ transport activity established with renal BBMVs (Fig. 3). The apparent affinities for P_i_ and Na^+^ were also in agreement with data from BBMV experiments, and the high expression in kidney, the signal being most prominent in cortex, concurred with P_i_ reabsorption along the renal proximal tubule (Figs. 2 and 3) [16, 23]. The faint band of about 5 kb with mRNA from lung on a northern blot probed with the human NaPi-IIa cDNA was the first sign of human NaPi-IIb, the “intestinal isoform”. Despite this appearance, it should take another 5 years until this cDNA was eventually cloned (Fig. 2b) [13].

An essential question remained unanswered, whether the identified gene/protein would respond to mediators of P_i_ homeostasis such as P_i_ availability and parathyroid hormone (PTH). An elegant strategy to test the specific contribution of NaPi-IIa to overall P_i_ transport in a mix of renal cortical RNA was adopted using RNaseH-mediated hybrid depletion [22]. Kidney RNA samples were hybridized with NaPi-II-specific DNA oligonucleotides and treated with RNase H, an endonuclease that specifically hydrolyses RNA hybridized to DNA. The hybrid-depleted samples were then analyzed by northern blotting and functional expression in *Xenopus* oocytes. To our delight, RNase H treatment significantly reduced both the NaPi-II-specific band on northern blots as well as P_i_ transport in injected oocytes. Moreover, we could show that a low P_i_ diet stimulated the abundance of NaPi-II mRNA and also P_i_ uptake in oocytes [32]. The so-called Hyp mouse suffers from a proximal tubular P_i_ reabsorption defect and proved an excellent system to further scrutinize the contribution of NaPi-II to maintaining P_i_ homeostasis. NaPi-IIa was indeed reduced in Hyp mice [30], though the Hyp gene was previously shown to encode a humoral factor (but not PTH) rather than the renal phosphate transporter itself [21]. PTH was found to have little effect on NaPi-II mRNA levels but to cause a striking reduction of transport protein at the apical membrane and an intracellular, punctate accumulation of the transporter [14]. The physiological role was further corroborated by the phenotypic characteristics of NaPi-IIa KO mice that showed renal P_i_ wasting, increased VitD_3_ levels, and a mild bone phenotype [3]. All these reports firmly established NaPi-IIa/Slc34A1 as a key player in balancing body P_i_ levels. Interestingly, it would take almost 10 years (and probably the invention of high-throughput DNA sequencing) until the first human mutations in *SLC34A1* (human NaPi-IIa) were identified in patient cohorts with renal stones [17, 25].

All these findings convincingly established the mechanism of P_i_ reabsorption at the apical membrane of renal proximal tubules; how P_i_ crossed the basolateral membrane to reach the renal interstitium remained an unsolved question. Recently, a promising candidate, Xpr1 (xenotropic and polytropic retroviral receptor 1), has been suggested [2, 8]. Shortly after cloning the apical transporter, we attempted to express a P_i_-efflux activity in oocytes by injecting proximal tubular mRNA, but failed. The injected oocytes were loaded with ^32^P, washed, and incubated in P_i_-free solution which was analyzed for leaked counts, though only insignificant amounts of radioactivity were detected. These experiments indicated that firstly, NaPi-II-mediated transport was unidirectional under standard uptake conditions (100 mM Na, 1 mM P_i_), and secondly, that an expression cloning approach would unlikely succeed in cloning “the basolateral transporter.”

## The laboratory

The Murer lab was a thriving environment, home to a mix of international young scientist from Italy (Simona Magagnin, Tiziano Verri), Spain (Juan Bertran, Manuel Palacin, and Victor Sorribas), Australia (Daniel Markovich), the USA (Chip Montrose, Steve Reshkin), Croatia (Branka Mrkic), Germany (Corinna Helmle-Kolb, Susanne Quabius, Gerti Stange), and Switzerland (Jutka Forgo, Andreas Werner, François Wuarin) including the more senior Juerg Biber and Heini Murer. The lab space was huge by current standards; we had dedicated rooms for RNA research, cloning, an oocyte room, a protein lab, an extended radioactive area, and even PhD students had proper office space. Quite outrageously according to current standards, smoking was permitted in offices and the coffee corner, also in the “protein lab” but not in DNA and RNA labs. The rationale was that “cigarette fumes could induce mutations when working with nucleic acids”—though it was not specified whether the samples or the researcher were at risk. The heart of the lab was arguably the coffee corner strategically placed between the two wings of the lab; passing it without interacting with other lab members could hardly be avoided usually resulting in scientific exchange and banter. The coffee corner also featured the latest issue of the daily national newspaper “Tages-Anzeiger” adding reference to the real world outside the lab. Importantly, the coffee corner was located next to the scintillation counter (probably in blatant violation of any current health and safety standards); this meant that everyone was immediately informed when a new clone was born. Since many guest scientists visited the laboratory in Zürich to learn and apply expression cloning, the “baptism” of new clones was quite frequent event. These successes were celebrated in style, with sparkling wine, Swiss cheese tartlets, and meaty delicacies and compensated for the long hours in the lab, the setbacks, and frustration with oocytes.

## Footnotes

1 Initially, the clones were named and numbered in order of appearance; NaPi-1, NaPi-2, NaPi-3 and so forth. The increasingly confusing mix of NaPis with clearly distinct structures from various species was later organized into the protein families NaPi-I (NaPi-1; Slc17A) and NaPi-IIa (NaPi-2 and NaPi-3, for example; Slc34A1), NaPi-IIb (NaPi-5; Slc34A2), NaPi-IIc (Slc34A3). The NaPi-III protein families includes the Na-dependent Pi cotransporters Pit1 and Pit2 (Slc20A1 and Slc20A2).
